# Simulation and Analysis of the Loading, Relaxation, and Recovery Behavior of Polyethylene and Its Pipes

**DOI:** 10.3390/polym16223153

**Published:** 2024-11-12

**Authors:** Furui Shi, P.-Y. Ben Jar

**Affiliations:** Department of Mechanical Engineering, University of Alberta, 10-203 Donadeo Innovation Centre for Engineering, 9211-116 Street NW, Edmonton, AB T6G 1H9, Canada; ben.jar@ualberta.ca

**Keywords:** relaxation, modeling, mechanical properties, polyethylene

## Abstract

Spring–dashpot models have long been used to simulate the mechanical behavior of polymers, but their usefulness is limited because multiple model parameter values can reproduce the experimental data. In view of this limitation, this study explores the possibility of improving uniqueness of parameter values so that the parameters can be used to establish the relationship between deformation and microstructural changes. An approach was developed based on stress during the loading, relaxation, and recovery of polyethylene. In total, 1000 sets of parameter values were determined for fitting the data from the relaxation stages with a discrepancy within 0.08 MPa. Despite a small discrepancy, the 1000 sets showed a wide range of variation, but one model parameter, $\sigma_{v,L}\left(0\right)$, followed two distinct paths rather than random distribution. The five selected sets of parameter values with discrepancies below 0.04 MPa were found to be highly consistent, except for the characteristic relaxation time. Therefore, this study concludes that the uniqueness of model parameter values can be improved to characterize the mechanical behavior of polyethylene. This approach then determined the quasi-static stress of four polyethylene pipes, which showed that these pipes had very close quasi-static stress. This indicates that the uniqueness of the parameter values can be improved for the spring–dashpot model, enabling further study using spring–dashpot models to characterize polyethylene’s microstructural changes during deformation.

## 1. Introduction

Polymers are widely used in our daily life [1,2], among which more than two-thirds are semi-crystalline polymers (SCPs) [3]. SCPs, such as polyethylene (PE), are a class of thermoplastics with complicated microstructures [4,5,6,7,8], which have attracted significant attention from many research groups [9,10,11,12,13,14,15,16,17]. In view of the fact that SCPs are increasingly used in various industrial sectors for fluid transportation [18], packaging [19], electronics [20], civil engineering [21], aerospace [22], medical devices [23], automotive components [24], etc., due to their chemical inertness and attractive mechanical properties [25,26,27,28,29], it is important to provide a proper characterization of their stress response to deformation. However, SCPs exhibit complex time-dependent behaviors, including relaxation and creep [4,30,31,32,33,34,35], which could significantly impact their performance in all applications. Therefore, a full characterization of SCPs for their mechanical behavior, which includes the time-dependent stress response to deformation, is essential to ensure reliable performance in their entire designed lifetime [36].

Stress relaxation under a constant deformation level has long been used to assess the performance of plastic pipes [37,38]. Moser and Folkman [38] demonstrated the usefulness of using stress relaxation tests to predict the long-term performance of plastic pipes and their interaction with soil systems [39]. In view of the fact that plastic pipes are designed to have a lifespan exceeding 50 years [40,41,42,43,44,45], with about 95% of plastic pipes made of PE [44,46,47,48,49], stress relaxation tests and the corresponding data analysis based on modeling have been widely used to study the long-term mechanical performance of PE and its pipes [44].

In a relaxation test at a constant deformation level, the stress decrease is very significant at the beginning, but eventually reaches an asymptotic limit [26,50]. The stress–time curve during the relaxation process is known to be influenced by the loading rate prior to relaxation [46], and a transition of the mechanism involved in the deformation process could be detected by characterizing the relaxation behavior before and after the transition [51,52]. Although the relaxation and recovery processes are known to give different stress responses to deformation, as the former is introduced after loading and the latter after unloading, both are carried out at a constant deformation level, with a bigger stress change in the former than in the latter [53]. At the same deformation level, the two processes are expected to reach the same stress level that is known as quasi-static stress. We have recently developed a test for characterizing SCPs’ viscous behavior, named the multiple-relaxation–recovery test (RR test), in which a recovery process is generated right after a relaxation process at a similar deformation level, and the two processes are repeated multiple times with the increase in specimen displacement [54,55]. Compared to the multiple-relaxation test described in the literature [51], the RR test allows for the determination of the unloading stiffness of the materials and reveals the unusual stress response of recovery behavior.

Various models have been used to analyze the mechanical test results of SCPs [56,57,58,59,60,61,62,63,64,65,66,67,68], among which models consisting of springs and dashpots have been used to mimic the stress response to deformation. Basic spring–dashpot models are known as Maxwell [69] and Voigt models, which represent the basic relaxation and creep behaviors, but are insufficient for simulating SCPs’ highly nonlinear behavior [64]. However, when Eyring’s equation was used to govern the stress response of the dashpot element [70,71,72,73,74,75,76,77], some success was obtained. Recently, the three-branch model proposed by Hong et al. [78,79] and Izraylit et al. [80], with only one branch containing an Eyring dashpot, was successfully used to mimic relaxation behavior. However, this model was not applicable to the recovery behavior after unloading [81]. Our recent work [81] also showed that some three-branch spring–dashpot models are not able to provide a full description of the stress change during relaxation and recovery phases of the RR test, especially for the unusual stress drop detected during the recovery. A three-branch model with two Maxwell branches and one spring branch, on the other hand, has been able to simulate both relaxation and recovery behavior fairly accurately. Most of the works using a three-branch model [82,83,84,85,86,87] only provided a single set of parameter values to mimic the experimental data, even though it is commonly believed that multiple sets of the parameter values exist for a model to mimic the experimental data [88,89,90]. As a result, the use of a spring–dashpot model to reproduce the experimental data is often considered merely a curve-fitting exercise. The parameter values were not used to characterize the viscous part of the mechanical properties of SCPs. Therefore, it is necessary to develop a novel approach to improve the uniqueness of model parameters for the accurate simulation and characterization of SCPs’ mechanical behavior. Such an approach is the subject of this paper.

In this work, an analysis method was developed based on global and local optimization to simulate the relaxation, recovery, and loading behaviors of PE and its pipes using a three-branch spring dashpot model based on Eyring’s law. The model contains two time-dependent, viscous branches and one time-independent, quasi-static branch. Data from RR tests on cylindrical specimens and notched pipe ring (NPR) specimens were used in the simulation to generate 1000 sets of parameter values to mimic the stress drop at the relaxation stages. The range of variation for these parameter values was examined and discussed. The best five fits were selected to improve the uniqueness of the model parameter values. Then, the analysis method was applied to four PE types of pipes, and their quasi-static stress as a function of specimen displacement was determined and discussed.

## 2. Experiments

### 2.1. Materials

**Figure 1:**
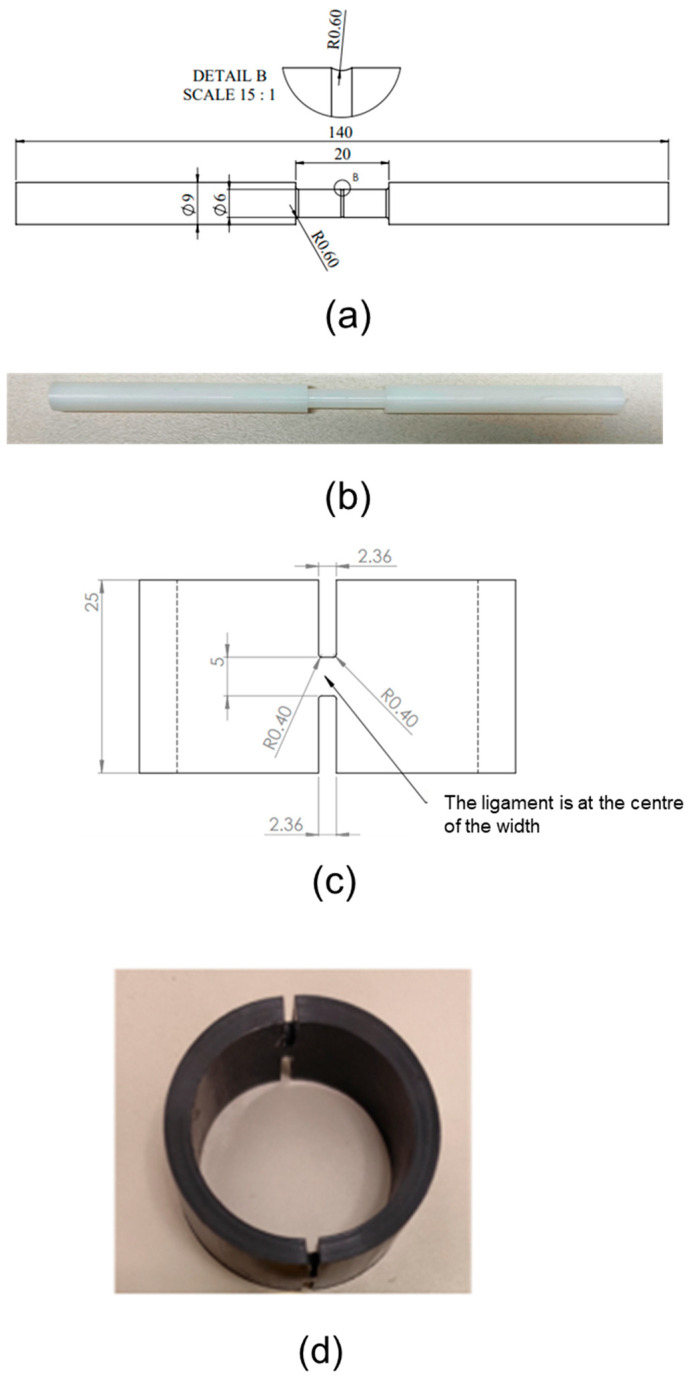
Specimens used in the RR tests: (**a**) dimensions of cylindrical specimen, (**b**) cylindrical specimen, (**c**) dimensions of NPR specimen (PE-Xa pipe as an example), and (**d**) NPR specimen. All units are in millimeters.

Cylindrical specimens of one type of HDPE [81] and NPR specimens of four different pipes were used in this study. The cylindrical specimen, named HDPE-b following the previous publication, has characteristics as detailed in the previous work [81]. The dimension of the cylindrical specimen is shown in Figure 1a, and Figure 1b shows the cylindrical specimen prepared for the tests. Figure 1c shows the dimensions of the NPR specimen cut from the PEX-a pipe, and a sample of the specimen is shown in Figure 1d. The four types of NPR specimens were obtained from four PE pipes, with their characteristics summarized in Table 1, which lists the materials of the four pipes, pipe name, density, yield strength, and hydrostatic design basis (HDB), defined in ASTM D2837 [91], representing the long-term hydrostatic strength of a pipe. All pipes have a ratio of pipe outer diameter to wall thickness (SDR) of 11.

The set-up of the RR test in the universal test machine was depicted in refs. [92,93,94,95,96].

### 2.2. Mechanical Characterization

RR tests were carried out using a Qualitest Quasar 100 universal test machine (Qualitest, Lauderdale, FL, USA), with data collected by a personal computer [51]. The details of the RR tests were described in the previous work [81,96]. The RR test consists of six stages in one cycle: 1st loading, relaxation, 2nd loading, stabilization, unloading, and recovery. The maximum deformation introduced in the RR tests was set to exceed the yield point, at which approximately 30 cycles were generated [51]. The sample curves of the RR tests on cylindrical specimens are available in previous publications [81,96]. The crosshead speed was set to 1 mm/min, with 10,000 s allocated for each relaxation, stabilization, or recovery stage. To ensure repeatability and reliability, two specimens were tested for each material, except for the PE4710-black pipe, for which only one RR test was conducted due to the laboratory shutdown in the COVID-19 pandemic period.

## 3. Data Analysis

### 3.1. Three-Branch Model

**Figure 2:**
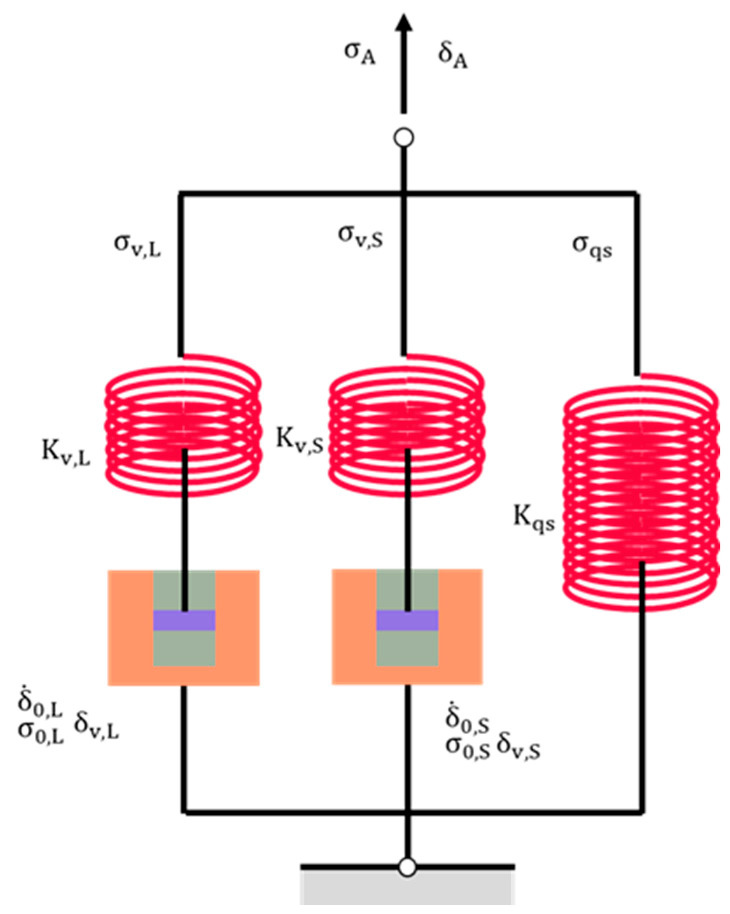
Three-branch spring–dashpot model used in this study.

In this study, the three-branch, spring–dashpot model employed for the simulation of the relaxation, recovery, and loading behaviors of the results from RR tests is depicted in Figure 2. This model is known as the Maxwell–Weichert model, which has been commonly used to mimic the stress response to deformation of a variety of materials [56,83,85,97,98,99,100]. As shown in Figure 2, the model incorporates three springs governed by Hooke’s law [52,101,102,103,104,105] and two dashpots governed by Eyring’s law [106,107,108,109,110,111,112,113]. The left, middle, and right branches represent long-term viscous stress, short-term viscous stress, and quasi-static stress, respectively, denoted by the subscripts L, S, and qs [114].

From our previous publication on the three-branch model [96], the equations governing stress response as a function of time during the relaxation, recovery, and loading stages were derived. The stress change [107] during each relaxation or recovery stage can be expressed as follows:

(1) $$\begin{array}{c} {∆\sigma }_{A}=\sigma_{A}(0)-\sigma_{A}(t)\\ =\sigma_{v,L}(0)+\sigma_{v,S}(0)\\ -2\sigma_{0,L}{tanh}^{-1}\{tanh[\sigma_{v,L}(0)/({2\sigma }_{0,L})]exp\;(-t/\tau_{v,L})\}\\ -2\sigma_{0,S}{tanh}^{-1}\{tanh[\sigma_{v,S}(0)/\left({2\sigma }_{0,S}\right)]\exp\left(-t/\tau_{v,S}\right)\} \end{array}$$

(2) $$\tau_{v,i}=\sigma_{0,i}/(K_{v,i}{\dot{\delta }}_{0,i})$$

where $\sigma_{A}$ represents the applied engineering stress, t the time from the beginning of the stage, $\sigma_{v,i}(0)$ the viscous stress at the beginning of the stage, $\sigma_{0,i}$ the reference stress, $\tau_{v,i}$ the characteristic relaxation time, $K_{v,i}$ the spring stiffness, and ${\dot{\delta }}_{0,i}$ the reference stroke rate, for i=L or S.

For each loading stage, the stress responses for the long-term and short-term branches were determined as follows:

(3) $${\dot{\sigma }}_{v,L}=K_{v,L}{\dot{\delta }}_{A}-(\sigma_{0,L}/\tau_{v,L})sinh(\sigma_{v,L}/\sigma_{0,L})$$

(4) $${\dot{\sigma }}_{v,S}=K_{v,S}{\dot{\delta }}_{A}-(\sigma_{0,S}/\tau_{v,S})sinh(\sigma_{v,S}/\sigma_{0,S})$$

where ${\dot{\delta }}_{A}$ is the crosshead speed of the test machine and ${\dot{\sigma }}_{v,i}$ is the first derivative of $\sigma_{v,i}$ with respect to time t, for i=L or S.

To estimate values for the fitting parameters in Equations (1), (3), and (4), the inverse analysis method [115,116,117,118,119,120,121,122,123,124,125,126,127] was employed by simulating the experimental data of the RR tests.

### 3.2. Method for Data Analysis

This section describes a new analysis method for the simulation of the relaxation, recovery, and loading behavior of PE and its pipes. The analysis method uses a new optimization approach that combines global and local optimization techniques.

In our previous work [96], a genetic algorithm (GA) in MATLAB R2021b was used to determine model parameter values via the inverse approach. However, that method was constrained by several assumptions that limited its applicability to a specific type of loading range. For example, the method depends on the presence of a plateau region [51] of the stress–displacement curve to determine one of the model parameter values. For test data that do not have such a clear plateau region, the method could not be used.

In the current study, a method was developed without the requirement of a plateau region. Rather, the new method focuses solely on the minimization of the maximum difference between the experimental data and values generated by the model in Figure 2, based on the principle known as minimax in approximation theory [128,129]. Setiyoko et al. [130] reported minimax as an approach that contrasts the widely used least squares for determining values for parameters [82,85,131,132,133,134,135,136,137,138]. Many researchers have typically determined a single set of values for their model parameters [139,140,141,142], but whether the values for the model parameters are unique remains a challenging question. In our previous work [96], ten sets of values for the model parameters in Figure 2 were determined to examine variations in the values [96]; however, the time for determining the ten sets of values was long due to constraints imposed in the algorithms, such as the assumption of the plateau region. By removing these assumptions, it became possible to obtain 1000 sets of the parameter values within a reasonable timeframe.

All programs developed in this study were coded in MATLAB R2024a, and the values for the model parameters in Equations (1), (3), and (4) were determined using experimental data at the relaxation, the recovery, and the first loading stages of the RR tests. At each of the relaxation or recovery stages, the values for the parameters in Figure 2 were assumed to remain fixed as the material microstructure during the relaxation and recovery was deemed to remain unchanged [143]. At each of the first loading stages, values for $K_{v,L}$ and $K_{v,S}$ were assumed to remain fixed as the deformation range introduced at each of the first loading stages was deemed to be small enough to allow the values for $K_{v,L}$ and $K_{v,S}$ to remain constant. However, the values for $\sigma_{0,L}$, $\tau_{v,L}$, $\sigma_{0,S}$, and $\tau_{v,S}$ were allowed to vary at each of the first loading stages.

**Figure 3:**
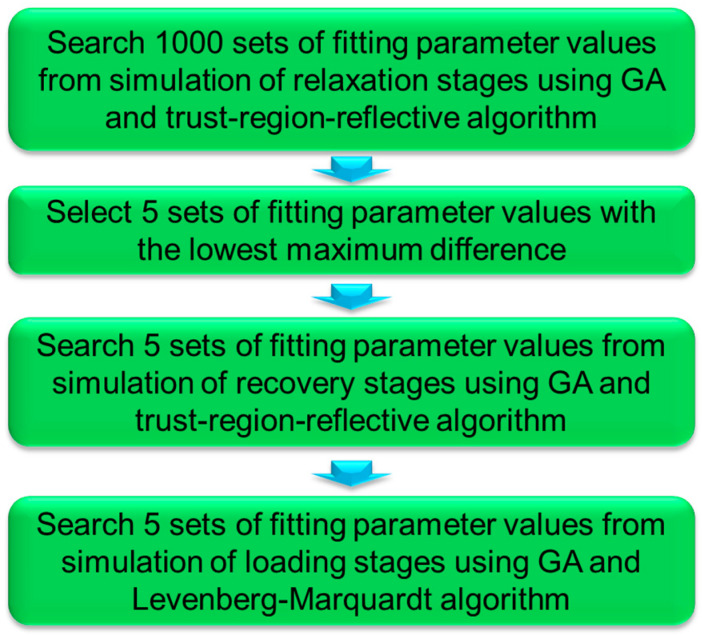
Procedure for the determination of fitting parameters in the relaxation, recovery, and loading stages of RR tests.

Figure 3 depicts the entire procedure used to determine values for the fitting parameters in Figure 2, including the initial 1000 sets of parameter values based on the experimental data at the relaxation stages, and then the 5 best sets of parameter values at each of the recovery and the 1st loading stages. The objective function of the analysis was to minimize the maximum difference between the experimental data and the values generated by the model.

The programs for identifying the optimal fitting parameter values are based on the hybrid combination of the global optimization approach, GA, and the local optimization approach, lsqnonlin [144,145,146,147] in MATLAB R2024a, also known as the combined two-phase strategy [148]. GA was first used to identify the fitting parameter values, and the generated fitting parameter values were set as the initial guesses of lsqnonlin. The objective function of GA is the maximum difference in stress response between the experiments and the model, which needs to be minimized. The population size was set to be 200, and the maximum number of generations was 600. According to Renders and Flasse [149], global optimization inherently involves a fundamental conflict between accuracy, reliability, and computing time. As a result, Mahinthakumar and Sayeed [150] suggested that the strength of GA could be decreased when the population was converged to a narrow location in the search space and the difference between solutions was small. It was also reported that GA often requires extensive iterations and tends to converge slowly [151,152,153]. On the other hand, local optimization is more efficient in narrow search areas and thus is increasingly hybridized with GA to accelerate computation [154,155,156,157,158]. As a result, a hybrid global–local approach was developed, by using GA and lsqnonlin [144,145,146,147] in MATLAB R2024a for global and local optimization [149], respectively, to identify the fitting parameters for the relaxation stages of the RR tests.

In the first step, as illustrated in Figure 3, a numerical method was developed using the inverse approach to search for 1000 sets of values for the fitting parameters in the three-branch model in Figure 2 in order to mimic the experimental data at the relaxation. The initial value ranges were set to be the same as those in the previous work [96], i.e., [0.1, 20] (in MPa) for $\sigma_{v,L}\left(0\right)$, [0.01, 2] (in MPa) for $\sigma_{0,L}$, [1000, 90,000] (in second) for $\tau_{v,L}$, [0.1, 20] (in MPa) for $\sigma_{v,S}\left(0\right)$, [0.01, 2] (in MPa) for $\sigma_{0,S}$, and [1, 900] (in second) for $\tau_{v,S}$. GA was used to identify the six fitting parameters in Equation (1), and the generated fitting parameter values were set as the initial guesses of lsqnonlin which were based on the trust-region-reflective algorithm [159]. In view of the fact that the speed of the computer program could be increased using parallel computing [160], parallel computing was implemented using ‘parfor’ in MATLAB R2024a, following the work in ref. [161], to speed up the simulation so that 1000 sets of model parameter values could be determined at the first step in a reasonable timeframe. In addition, the experimental data for the very first relaxation stage were ignored in the analysis, because it did not have any prior recovery stage, and thus did not possess the same deformation history as the relaxation stages in other cycles. In other words, the analysis conducted in this study always started from the relaxation stage in the second cycle of the RR test.

In the second step of Figure 3, five sets of fitting parameter values with the smallest maximum difference between the experimental data at the relaxation stages and the simulation results were selected. In the third step, each of the five sets of values from the second step was used to determine one set of fitting parameter values for the recovery stages at similar deformation levels. The initial values of the fitting parameters at the recovery stages, for example in the mth cycle of the RR tests, were set to be [0.01, α] (in MPa) for $\sigma_{v,L}\left(0\right)$, [0.001, β] (in MPa) for $\sigma_{0,L}$, [1000, 90,000] (in second) for $\tau_{v,L}$, [−20, −0.001] (in MPa) for $\sigma_{v,S}\left(0\right)$, [0.001, 2] (in MPa) for $\sigma_{0,S}$, and [1, 10,000] (in second) for $\tau_{v,S}$, where α and β are the values for $\sigma_{v,L}\left(0\right)$ in the relaxation stage of the mth cycle and the $\sigma_{0,L}$ values in the next relaxation stage, i.e., in the (m+1)th cycle. In view of the fact that the range of stress variation at the recovery stages was much less than that at the corresponding relaxation stages, it was deemed unnecessary to determine 1000 sets of parameter values for the simulation of the recovery stages.

The final step in Figure 3 is to determine five sets of fitting parameter values for Equations (3) and (4) to simulate the stress variation at the 1st loading stage in each cycle, based on the parameter values determined for the relaxation and recovery stages in steps 2 and 3, respectively. For this purpose, the method was similar to that used in our previous work [96], based on GA in MATLAB R2021b, but with the improvement of combining GA with lsqnonlin. However, in this case lsqnonlin automatically employed the Levenberg–Marquardt algorithm, as the original method [96] was designed to fit only one data point at a time but the trust-region-reflective algorithm requires the number of data points (equations) to be at least equal to the number of parameters (variables).

It should be noted that in the literature, many researchers [51,52,92,95] have used constant characteristic relaxation time for their simulation. However, as suggested in ref [162], the effect of the characteristic relaxation time on the determination of $\sigma_{qs}$ should be evaluated and the characteristic relaxation time should be allowed to vary with deformation. The novelty of the proposed method, as described above, originates from its ability to allow change in the characteristic relaxation time during the deformation. The proposed method also enables the evaluation of the influence of the characteristic relaxation time on the determination of other model parameter values. In addition, the combination of global and local optimization also significantly reduced the searching time for the 1000 parameter values, allowing the selection of the best five sets of parameter values and thus evaluating the uniqueness of the parameter values for the characterization of the viscous behavior of SCPs.

### 3.3. Resolution of the Experimental Measurements

Many researchers have studied material properties using mechanical tests [108,141,163,164,165,166,167,168,169], but few have considered the resolution of the test data [170]. For example, Mulliken and Boyce [171] successfully predicted the stress response of polymers in tension and compression tests using a constitutive model, but the resolution of the experimental measurements was not reported to justify the quality of the prediction. According to Jar [165,172], the uncertainty of the experimental measurements affects the accuracy of the test results. Therefore, a model that provides a good fitting to the experimental data with a poor resolution does not provide a clear indication on the validity of the model. In view of this potential issue, the resolution of the stress measurements obtained from this study was determined to assess the accuracy of the test results.

For the cylindrical specimens, $\sigma_{A}$ was calculated using the following expression:

(5) $$\sigma_{A}=\frac{4F}{\left(\pi D^{2}\right)}$$

where F is the measured tensile force using the universal testing machine, and D the initial diameter of the gage section measured using a digital caliper. Therefore, the resolution of $\sigma_{A}$ for the cylindrical specimens, $d\sigma_{A}$, can be expressed as follows [170,173]:

(6) $$d\sigma_{A}=\left|\frac{4dF}{\left(\pi D^{2}\right)}\right|+\left|\frac{8FdD}{\left(\pi D^{3}\right)}\right|$$

where *dF* and *dD* are the resolutions of the force and diameter measurements, respectively.

Similarly, the resolutions for the NPR specimens can be calculated using the following equation.

(7) $$d\sigma_{A}=\left|\frac{dF}{t_{1}w_{1}{+t}_{2}w_{2}}\right|+\left|\frac{Fw_{1}dt_{1}}{{\left(t_{1}w_{1}{+t}_{2}w_{2}\right)}^{2}}\right|+\left|\frac{Ft_{1}dw_{1}}{{\left(t_{1}w_{1}{+t}_{2}w_{2}\right)}^{2}}\right|+\left|\frac{Fw_{2}dt_{2}}{{\left(t_{1}w_{1}{+t}_{2}w_{2}\right)}^{2}}\right|\;+\left|\frac{Ft_{2}dw_{2}}{{\left(t_{1}w_{1}{+t}_{2}w_{2}\right)}^{2}}\right|$$

where $t_{j}$ is the initial thickness of the gauge section j of the NPR specimens, and $w_{j}$ is the corresponding initial width of the gauge section (*j* is 1 or 2, representing the two ligaments of the NPR specimens).

In this study, the resolution of the universal test machine for the force measurement was 0.5 N and the resolution of the digital caliper for the dimensional measurement was 0.01 mm. As an example, for a cylindrical specimen with *D* of 5.90 mm and the maximum force of 402.5 N, the resolution of its stress measurement, $d\sigma_{A}$, is

(8) $$d\sigma_{A}=\left|\frac{4dF}{\left(\pi D^{2}\right)}\right|+\left|\frac{8FdD}{\left(\pi D^{3}\right)}\right|=0.0682\;MPa$$

Similarly, the resolution for the stress measurement of NPR specimens from different PE pipes can be determined based on the dimensions and maximum force generated in the RR tests.

## 4. Results and Discussion

### 4.1. Accuracy of the Simulation

This section presents 1000 sets of parameter values for the simulation of the relaxation stages in the RR tests, including the maximum difference between the simulation and the experimental data and a comparison of the simulation results with the resolution of the experimental data.

In the previous study, we found that the three-branch model can accurately describe results at the relaxation, recovery, and loading stages of RR tests [96]. The previous analysis relied on several assumptions, such as constant $\tau_{v,L}$ and $\tau_{v,S}$ values [52,174], and considered the continuity of the parameter values with the increase in deformation. In this study, the method presented in Section 3.2 was used to generate 1000 sets of parameter values for the simulation of experimental data at the relaxation stages of the RR tests on cylindrical specimens and NPR specimens. Table 2 summarizes the resolution of the measured stress data and their maximum difference with the modeling results, the latter based on the 1000 sets of fitting parameter values.

Table 2 shows the values for the experimental resolution based on Equations (6) and (7) and the maximum difference in stress between the experimental data and the simulation data using the 1000 sets of parameter values for cylindrical and NPR specimens at the relaxation stages. This indicates that the values of experimental resolution are slightly larger than the values of maximum difference in stress between the experimental data and simulation data. From Table 2, it should be noted that the values of the maximum difference are less than 0.07 MPa, which is smaller than 0.08 MPa reported in our previous work [96]. In the literature, the maximum difference between the experiments and model was reported to be in the range from 0.17 to about 1 MPa [30,85,175,176,177,178]. In addition, the difference between the resolution of the test data and the value for the maximum difference is less than 0.01 MPa, with the maximum difference in HDPE-b being even smaller than the resolution of the test data. This indicates that the analysis method created in this study can provide good agreement between the model and experiments. This high accuracy was also achieved for the NPR specimens in Table 2, with the maximum differences being less than 0.08 MPa.

The results in Table 2 show the capability of the three-branch model based on the proposed analysis method presented in Section 3.2, which is consistent with the work in the literature [99]. Jar [179] further validated the close simulation of the three-branch model in a new test, named the MR test, which entails relaxation behavior at different deformation levels. However, none of the results in these works found the maximum difference between the experiments and model to be less than 0.08 MPa. Table 2 also suggests that since the inverse approach relies on the quality of the experimental measurements, further improvement of the simulation accuracy requires the improvement of the resolution for the experimental data.

**Figure 4:**
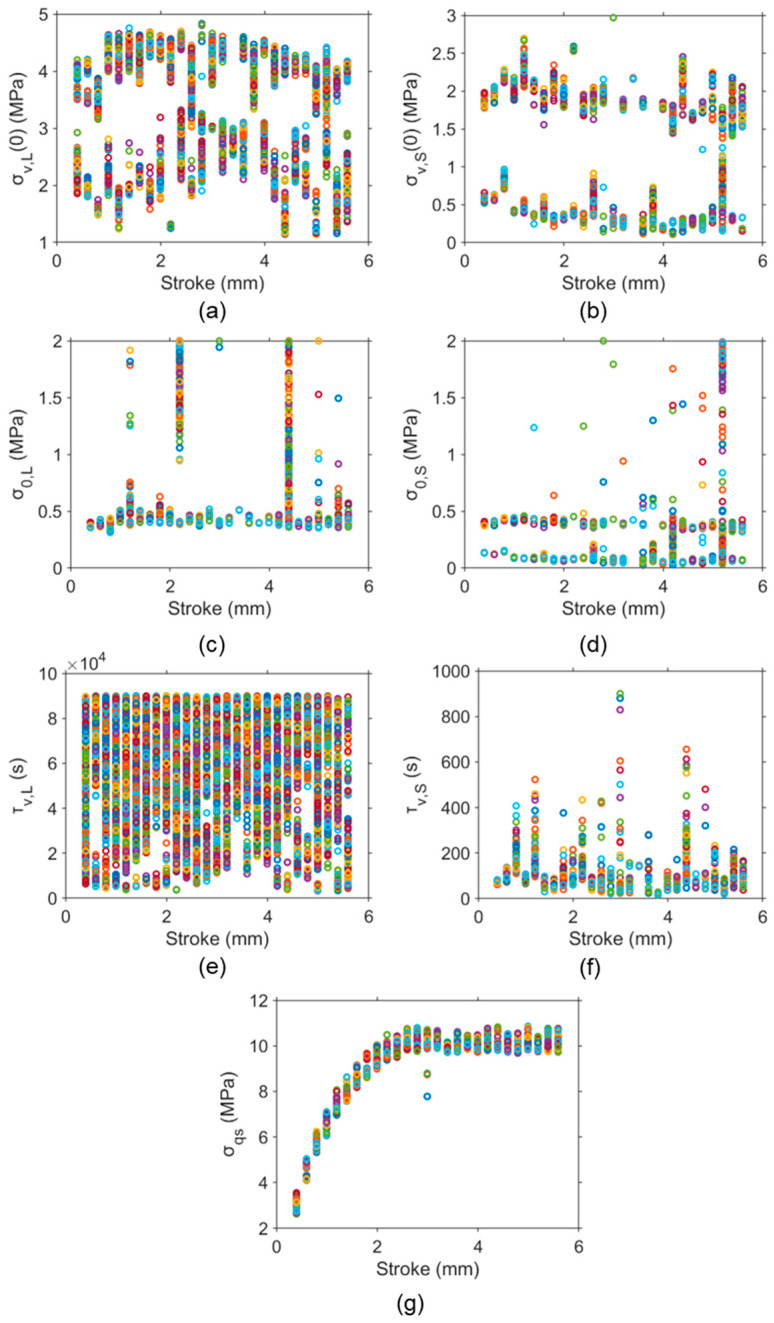
The 1000 sets of parameter values for simulation at the relaxation stages of different deformation levels in one RR test of HDPE-b: (**a**) $\sigma_{v,L}(0)$, (**b**) $\sigma_{v,S}(0)$, (**c**) $\sigma_{0,L}$, (**d**) $\sigma_{0,S}$, (**e**) $\tau_{v,L}$, (**f**) $\tau_{v,S}$, and (**g**) $\sigma_{qs}$. Different colors at one stroke are used to indicate the 1000 sets of parameter values.

Figure 4 illustrates 1000 sets of fitting parameter values for the simulation of the relaxation stages of different deformation levels of one RR test on an HDPE-b cylindrical specimen. As shown in Figure 4a, $\sigma_{v,L}(0)$ clearly follows two distinct paths with the increase in stroke, namely, an upper path and a lower path. Works in the literature always present a single path of the fitting parameters [92,108,174], even for our previous work, which showed ten sets of the fitting parameter values [96]. Note that Pyrz and Zaïri [180] identified 20 sets of parameter values but no pattern was identified for these values.

Figure 4 also suggests that a two-path pattern exists for the variation in $\sigma_{v,S}(0)$ and $\sigma_{0,S}$ with stroke in Figure 4b and Figure 4d, respectively, though $\sigma_{0,L}$ in Figure 4c mainly shows a single path. With the consideration of the limited resolution for the experimental measurement, this two-path pattern for $\sigma_{v,L}(0)$, $\sigma_{v,S}(0)$, and $\sigma_{0,S}$ values indicates that the fitting parameters could show some identifiable variation with the increase in deformation, rather than the random distribution that has been believed in the past. Therefore, there is a possibility that these model parameters could be linked to microstructural changes in SCPs.

For $\tau_{v,L}$ and $\tau_{v,S}$ values, as shown in Figure 4e,f, their values are scattered across the deformation levels considered in the RR test, indicating that variations in $\tau_{v,L}$ and $\tau_{v,S}$ values may not affect the two-path pattern for the fitting parameters $\sigma_{v,L}(0)$, $\sigma_{v,S}(0)$, and $\sigma_{0,S}$. These results confirm the previous suggestion that inaccurate values for the characteristic relaxation time have a minor influence on the simulation [167]. In the literature, the characteristic relaxation time was often fixed as a constant for different deformation levels and materials [51,174,181]. Although Izraylit et al. [80] determined the values of characteristic relaxation time at different deformation levels, they did not clearly present the curve-fitting process used in their study. Jar [179] obtained values of characteristic relaxation time as functions of deformation levels but only provided one set of fitting parameters.

Even with a two-path distribution for some of the fitting parameters, a single trend of variation with stroke could be established for $\sigma_{qs}$, as shown in Figure 4g. The band of variation for $\sigma_{qs}$ is quite small, suggesting that the $\sigma_{qs}$ values are not sensitive to the variation in the fitting parameter values. These findings suggest that the determination of $\sigma_{qs}$ does not require a unique set of values for the fitting parameters, as long as the fitting parameter can provide a reasonable simulation of the test results.

**Figure 5:**
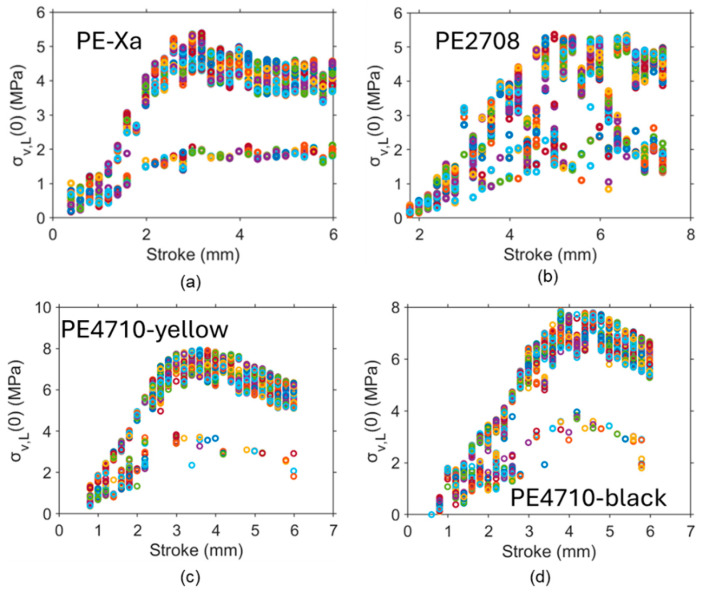
A two-path pattern of $\sigma_{v,L}(0)$ as a function of stroke for NPR specimens based on 1000 sets of parameter values: (**a**) PE-Xa, (**b**) PE2708, (**c**) PE4710-yellow, and (**d**) PE4710-black pipes. Different colors at one stroke are used to indicate the 1000 sets of parameter values.

Figure 5 summarizes the $\sigma_{v,L}(0)$ values of the pipe specimens, which clearly shows that a two-path pattern also exists for PE-Xa, PE2708, PE4710-yellow, and PE4710-black pipes, suggesting that the presence of two distinct paths for variations in $\sigma_{v,L}(0)$ with deformation is a common phenomenon. Figure 5 also shows that $\sigma_{v,L}(0)$ increases significantly at the early stage of the RR test, which is consistent with the observations reported in the literature [92]. Note that in the literature, Liu et al. [182], and Moore et al. [183,184,185] also compared modeling and experimental testing for the stress response of HDPE pipes, but they did not provide the viscous stress component of the stress response. Zhang and Jar [181] determined the viscous stress in the pipes but with the assumption that the characteristic relaxation time should be kept constant.

The above findings suggest that it is possible to improve the uniqueness of model parameter values which could be used to characterize the mechanical performance of SCPs. However, a further study would be needed to confirm this possibility.

### 4.2. Best Five Fits

One of the main problems addressed in the literature about the deformation of SCPs is the evolution of the crystalline phase with an increase in deformation [3]. Therefore, if the fitting parameters are to be used to characterize a material’s performance, the change in the fitting parameter values should reflect the evolution of SCP microstructures.

**Figure 6:**
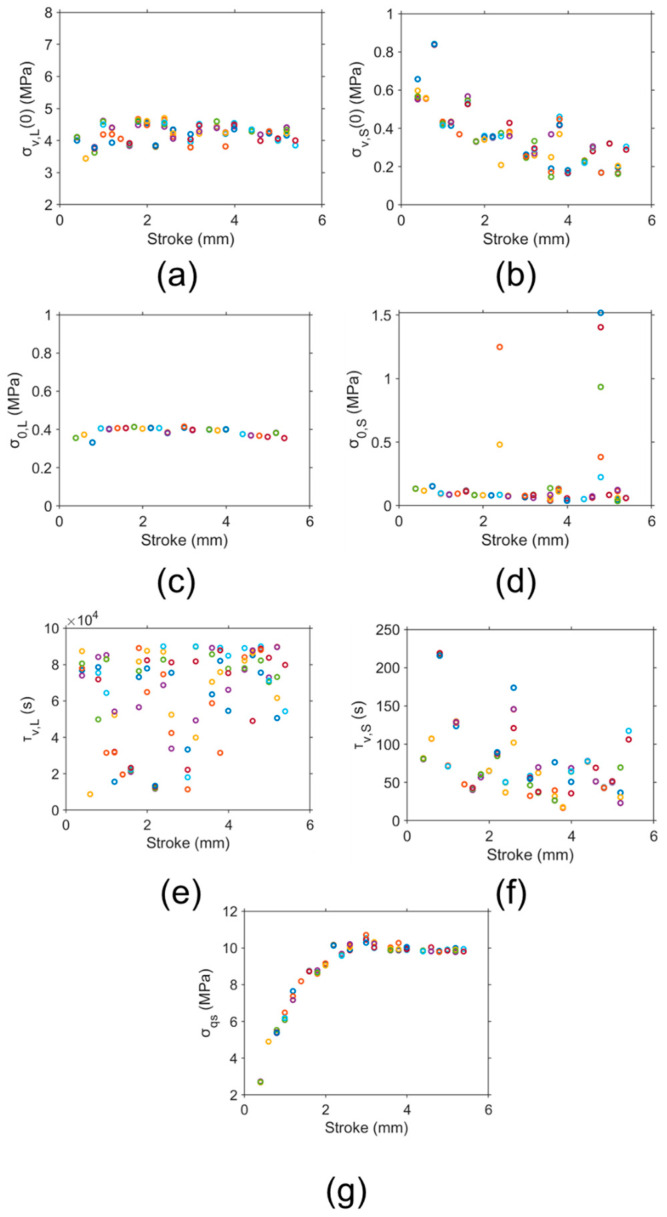
Best five sets of parameter values (in open red circles) selected from 1000 sets for the simulation of stress variation at the relaxation stages of HDPE-b and the corresponding $\sigma_{qs}$: (**a**) $\sigma_{v,L}(0)$, (**b**) $\sigma_{v,S}(0)$, (**c**) $\sigma_{0,L}$, (**d**) $\sigma_{0,S}$, (**e**) $\tau_{v,L}$, (**f**) $\tau_{v,S}$, and (**g**) $\sigma_{qs}$. Different colors at one stroke are used to indicate the five best sets of parameter values.

Many researchers [82,85] minimized the difference between the model and experiments to determine the model parameter values. Although the 1000 sets of parameter values are equally valid solutions of the model, because the uniqueness of the parameter values is absent among the 1000 sets, the five best sets of fitting parameter values were considered. Using the procedure depicted in Figure 3, five sets of fitting parameter values were identified which provided the closest simulation of the stress variation at the relaxation stages. These fitting parameter values for HDPE-b, along with its $\sigma_{qs}$, are summarized in Figure 6 as functions of stroke. Note that some outliers exist, especially for $\sigma_{v,S}(0)$ and $\sigma_{0,S}$, but apart from these outliers, a general trend for $\sigma_{v,L}(0)$, $\sigma_{v,S}(0)$, $\sigma_{0,L}$, and $\sigma_{0,S}$ values is clearly given with the increase in stroke.

It should be pointed out that the five sets of parameter values shown in Figure 6 gave the maximum difference in stress response to deformation between the simulation and the experimental data of less than 0.04 MPa at the relaxation stages. In view of the fact that these values are significantly smaller than the resolution of the experimental measurements of 0.0682 MPa, as shown in Table 2, a further study using a test set-up that gives a better resolution than that in the current study would be needed to verify the validity of the five sets of parameter values. Nevertheless, Figure 6 clearly shows that fitting parameter values with a clear trend of dependence with deformation could be determined using the proposed approach for the data analysis.

It should also be noted that the $\sigma_{v,L}(0)$ values in Figure 6a were located along the upper path in Figure 4a, which indicates that the best five sets improved the uniqueness of the values of the long-term viscous stress at the beginning of relaxation. In the literature, Sweeney et al. [85] also described the long-term relaxation behavior using a Maxwell model, but the uniqueness of the model parameter values was not considered.

Figure 6b shows $\sigma_{v,S}(0)$ values for the five best sets of fitting parameters. These $\sigma_{v,S}(0)$ values are much smaller than their $\sigma_{v,L}(0)$ counterpart in Figure 6a, which is consistent with the values determined before by the manual curve fitting [179].

The five sets of $\sigma_{0,L}$ values shown in Figure 6c indicate that the five values at a given stroke are very consistent, and are in the value range consistent with those obtained previously [51] using a different test method (MR test). Figure 6d presents the $\sigma_{0,S}$ values, showing that apart from these outliers, their values are smaller than the corresponding $\sigma_{0,L}$ values at the same stroke, consistent with the previous observations [179]. The values of $\sigma_{0,L}$ and $\sigma_{0,S}$ in Figure 6c,d are consistent with the values reported in the literature [51,92,174].

The $\tau_{v,L}$ and $\tau_{v,S}$ values shown in Figure 6e,f show significant scattering with the increase in stroke, though the $\tau_{v,S}$ values are smaller than the $\tau_{v,L}$ values. This implies that the $\tau_{v,L}$ values and $\tau_{v,S}$ values exhibited high variability. However, the scattering $\tau_{v,L}$ and $\tau_{v,S}$ values did not affect the consistency of the corresponding fitting parameters $\sigma_{v,L}(0)$, $\sigma_{v,S}(0)$, $\sigma_{0,L}$, and $\sigma_{0,S}$. This aligns with the findings in the literature that the values of the characteristic relaxation time play a minor role in the simulation [167].

Figure 6g shows the $\sigma_{qs}$ values as a function of stroke, which are consistent with values reported previously based on a different curve-fitting approach [81]. The figure suggests that $\sigma_{qs}$ values increase initially and then reach a plateau, consistent with the trend observed previously [51]. As expected, even with the significant scattering of $\tau_{v,L}$ and $\tau_{v,S}$ values in Figure 6e,f, some outliers for $\sigma_{0,S}$ in Figure 6d, and some scattering for $\sigma_{v,L}\left(0\right)$ and $\sigma_{v,S}\left(0\right)$ in Figure 6a and 6b, respectively, the five sets of $\sigma_{qs}$ values are still very consistent. In view of the measurement resolution shown in Table 2, this suggests that the $\sigma_{qs}$ values determined from the current method have high consistency, not much affected by variations in fitting parameter values determined by the inverse approach.

It should be noted that, although some scattering is present, the consistency of the fitting parameter values shown in Figure 6 is much better than that reported in the literature. For example, Xu et al. [88] developed a generalized reduced gradient optimization algorithm, and used the algorithm to determine the parameter values for a three-branch model. Their results showed a much more significant scattering than those shown in Figure 6. Therefore, the proposed analysis method can capture a much more appropriate set of parameter values for the characterization of SCPs than the approaches currently available in the literature.

Table 3 lists the best five sets of fitting parameters for HDPE-b at the relaxation stage around the yield point. It was found that the five sets of $\sigma_{0,L}$ values are nearly identical to each other. In the literature, Xu et al. [88] determined three sets of model parameter values and the coefficient of variation was more than 50%. This indicates that the best-five-fits method in this study could provide better model parameter values than theirs. It was also found that the $\sigma_{v,L}\left(0\right)$ values are higher than the $\sigma_{v,S}\left(0\right)$ values, which is consistent with the results reported in the literature [179].

**Figure 7:**
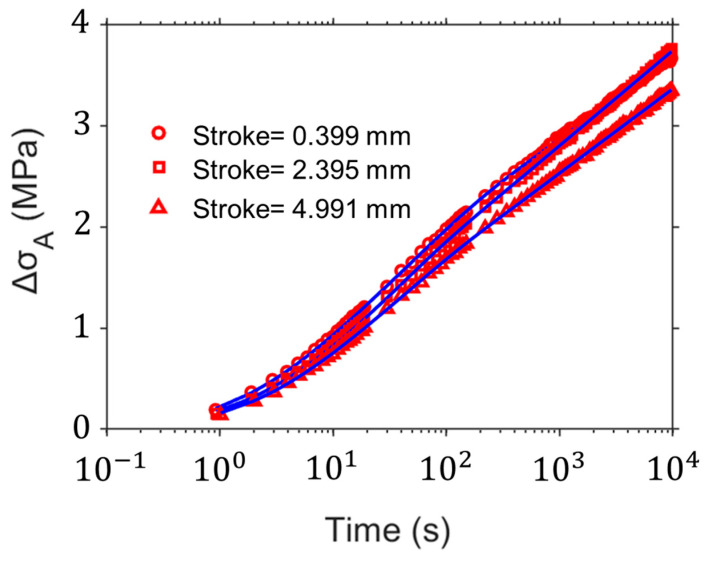
Simulation of stress change at relaxation stages of different strokes for HDPE-b using the fitting parameter values in Figure 6.

Figure 7 shows the simulation of the stress change in relaxation stages at different strokes using the fitting parameter values from the best five sets in Figure 6. The symbols in Figure 7 represent the experimental data and the lines represent the simulation data. This indicates that the parameter values determined from the current method can provide a quite accurate description of the relaxation behavior.

**Figure 8:**
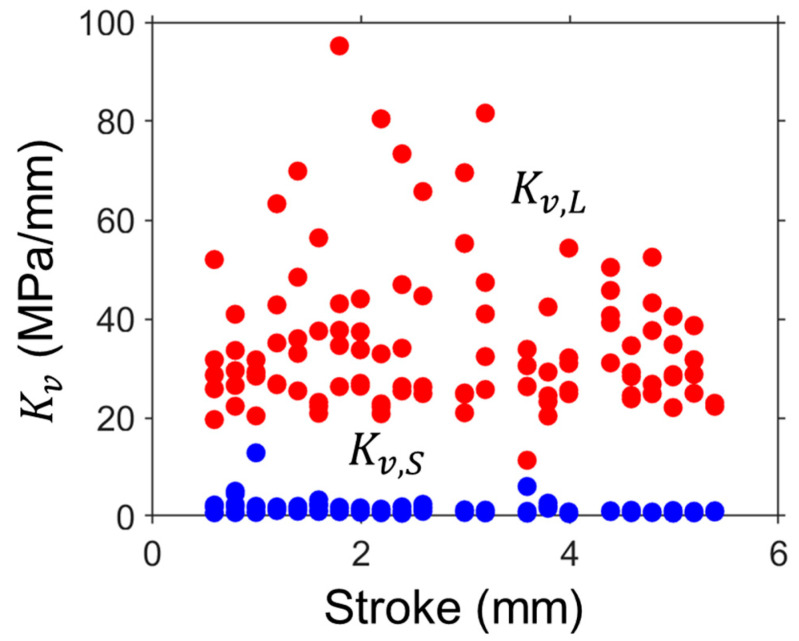
$K_{v,L}$ and $K_{v,S}$ as a function of stroke of HDPE-b.

Figure 8 shows the $K_{v,L}$ and $K_{v,S}$ values of HDPE-b as functions of stroke. Figure 8 suggests that most of the $K_{v,L}$ values are higher than the $K_{v,S}$ at the same stroke. Note that the difference between $K_{v,L}$ and $K_{v,S}$ values has been an open question in the literature, as the works reported indicate that $K_{v,L}$ values could be either larger or smaller than $K_{v,S}$ [83,88,179]. This uncertainty could be explained by the results presented in Figure 4, as $K_{v,L}$ values are influenced by the choice of $\sigma_{v,L}(0)$ values from the two paths in Figure 5a. When the lower path in Figure 4a is used to determine $K_{v,L}$, in view of the fact that the corresponding $\sigma_{v,S}(0)$ values belong to the upper path in Figure 4b, $K_{v,S}$ must be larger than $K_{v,L}$. Conversely, the $K_{v,L}$ values are larger than $K_{v,S}$. As shown in Figure 8, for the best five sets of fitting parameter values, the $\sigma_{v,L}(0)$ values belong to the upper path. Therefore, the $K_{v,L}$ values for HDPE-b should be larger than the $K_{v,S}$ values. The above explanation is based on the identification of the two-path pattern for $\sigma_{v,L}(0)$ and $\sigma_{v,S}(0)$, which would not be possible without the collection of a large number of fitting parameter values (1000 sets). Similarly, it was found that the $K_{v,L}$ values are higher than the $K_{v,S}$ for pipes in this study.

In addition, we believe that the $K_{v,L}$ and $K_{v,S}$ values could represent the microstructural changes in PE during the deformation process [179]. The accurate determination of $K_{v,L}$ and $K_{v,S}$ values is essential for examining the possible relationship between microstructural changes and mechanical performance of SCPs. This study provides an approach that could clearly distinguish the difference between $K_{v,L}$ and $K_{v,S}$ values, which has not been possible using other approaches reported in the literature.

**Figure 9:**
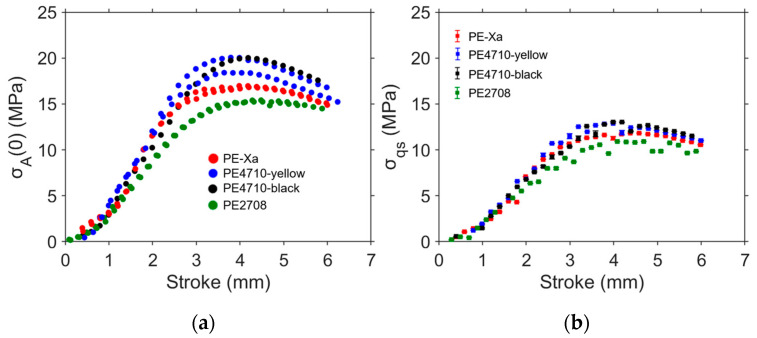
Summary RR test results for NPR specimens: (**a**) applied stress at the onset of relaxation,$\;\sigma_{A}$(0), and (**b**) $\sigma_{qs}$.

Figure 9 compares $\sigma_{A}$(0) and $\sigma_{qs}$ for NPR specimens from the four pipes in Table 2. Markers in Figure 9b represent the average of the five $\sigma_{qs}$ values that were determined based on the five sets of the best-fitting parameter values using the procedure described in Figure 3. The error bars in Figure 9b depict the standard deviation of the five $\sigma_{qs}$ values. It was found that although the $\sigma_{A}$(0) values for PE-Xa and PE2708 are lower than those for PE4710-yellow and PE4710-black at the same stroke, their $\sigma_{qs}$ values are much closer to each other.

## 5. Conclusions

This paper presents a new analysis method based on global and local optimization for the simulation of the relaxation, recovery, and loading behaviors of PE and its pipes in RR tests on cylindrical and NPR specimens, respectively. The results from the RR tests can be accurately mimicked using the three-branch model with the parameter values determined using the proposed analysis approach, and the maximum difference between the stress measured experimentally and that determined from the model is much smaller than the values reported in the literature.

Based on the proposed analysis method, 1000 sets of fitting parameter values were determined to simulate stress variations at the relaxation stages at different deformation levels, with the discrepancy between the experimental data and simulation results below 0.08 MPa. The $\sigma_{v,L}(0)$ values show two distinct paths with the increase in the stroke. The best five sets selected from the 1000 sets of parameter values provide a closer simulation of the relaxation behavior with a maximum difference in the stress response of less than 0.04 MPa. The results from the best five fits show that the proposed method can determine consistent values and a clear trend for $\sigma_{v,L}(0)$, $\sigma_{v,S}(0)$, $\sigma_{0,L}$, and $\sigma_{0,S}$. The results also indicate that the analysis method is better than any of the methods reported in the literature on the parameter identification of spring–dashpot models. The results from this study suggest that it is possible to improve the uniqueness of parameter values, which can then be used to characterize the viscous component of mechanical behavior for SCPs. This study also confirms that the $K_{v,L}$ values for PE should be larger than the $K_{v,S}$ at the same stroke, which solved the problem of uncertainty of the relationship between $K_{v,L}$ and $K_{v,S}$ in the literature. The results from the simulation suggest that variations in the characteristic relaxation time do not have much influence on the variations in other fitting parameter values, which confirms the previous finding that the effect of characteristic relaxation time has little influence on the variations in other fitting parameter values. However, further study is needed to improve the resolution of the measured results, so that the accuracy of the values based on the best five sets of fitting parameter values can be verified.

The overall conclusions of this study are as follows. The uniqueness of parameter values can be improved, except for the characteristic relaxation time, as the characteristic relaxation time has a minor influence on the modeling. On the other hand, if the experimental data have a sufficiently high resolution to reduce the uncertainty of the test results, it is then possible to explore the relationship between these parameters and microstructural changes in polyethylene during the deformation using the proposed method. This study provides a tool to improve the uniqueness of the model parameter values in a three-branch model.

## Figures and Tables

**Table 1:** Characteristics of pipes used in this study.

Material	Pipe Name	Density (kg/m^3^)	Yield Strength (MPa)	HDB @23 °C (MPa)
HDPE	PE4710-black	949	24.8	11.03
HDPE	PE4710-yellow	949	>24.1	11.03
PEX	PE-Xa	938	19	8.62
MDPE	PE2708	940	19.3	8.62

**Table 2:** Resolution of the measured stress data and maximum difference in the stress response between the experiments and model during relaxation stages based on the 1000 sets of model parameter values.

Sample Specimens	Resolution of Experimental Measurement (MPa)	Max Difference of Stress Between Experimental Measurements and Model Simulation from the Study (MPa)
HDPE-b, cylindrical	0.0682	0.0618
PE-Xa, NPR pipe	0.0767	0.0759
PE4710-yellow, NPR pipe	0.0746	0.0666
PE4710-black, NPR pipe	0.0743	0.0591
PE2708, NPR pipe	0.0590	0.0524

**Table 3:** Best five sets of parameter values around the yield point for HDPE-b.

Model Parameters	Set 1	Set 2	Set 3	Set 4	Set 5
$\sigma_{v,S}(0)$ (MPa)	0.26	0.27	0.33	0.29	0.30
$\sigma_{0,S}$ (MPa)	0.06	0.06	0.09	0.08	0.08
$\tau_{v,S}$ (s)	62.51	69.78	36.70	37.80	37.88
$\sigma_{v,L}(0)$ (MPa)	4.21	4.28	4.47	4.51	4.47
$\sigma_{0,L}$ (MPa)	0.40	0.40	0.40	0.40	0.40
$\tau_{v,L}$ (s)	39,881.24	49,320.51	89,792.94	89,999.71	81,756.19

## Data Availability

The data supporting the findings described in this manuscript are available from the corresponding authors upon request.
